# Global Assessment of *Antrodia cinnamomea*-Induced MicroRNA Alterations in Hepatocarcinoma Cells

**DOI:** 10.1371/journal.pone.0082751

**Published:** 2013-12-17

**Authors:** Yen-Ju Chen, Mike W. C. Thang, Yu-Tzu Chan, Yu-Feng Huang, Nianhan Ma, Alice L. Yu, Chung-Yi Wu, Miao-Lin Hu, Kuo Ping Chiu

**Affiliations:** 1 Genomics Research Center, Academia Sinica, Taipei, Taiwan; 2 Genome and Systems Biology Degree Program, National Taiwan University, Taipei, Taiwan; 3 Center of Stem Cell and Translational Cancer Research, Chang Gung Memorial Hospital, Taoyuan, Taiwan; 4 Institute of Systems Biology and Bioinformatics, National Central University, Jhongli, Taiwan; 5 Center of Stem Cell and Translational Cancer Research, Chang Gung Memorial Hospital and Chang Gung University, Taoyuan, Taiwan; 6 Department of Food Science and Biotechnology, National Chung Hsing University, Taichung, Taiwan; 7 Department of Life Sciences, College of Life Sciences, National Taiwan University, Taipei, Taiwan; The University of Tennessee Health Science Center, United States of America

## Abstract

Recent studies have demonstrated a potent anticancer potential of medicinal fungus *Antrodia cinnamomea*, especially against hepatocarcinoma. These studies, however, were performed with prolonged treatments, and the early anticancer events remain missing. To probe the early anticancer mechanisms of *A. cinnamomea*, we treated SK-Hep-1 liver cancer cell with *A. cinnamomea* fruiting body extract for 2 and 4 hours, sequenced RNA samples with next-generation sequencing approach, and profiled the genome-wide miRNA and mRNA transcriptomes. Results unmistakably associated the early anticancer effect of *A. cinnamomea* fruiting body extract with a global downregulation of miRNAs which occurred solely in the *A. cinnamomea* fruiting body extract-treated SK-Hep-1 cells. Moreover, the inhibitory effect of *A. cinnamomea* fruiting body extract upon cancer miRNAs imposed no discrimination against any particular miRNA species, with oncomirs miR-21, miR-191 and major oncogenic clusters miR-17-92 and miR-106b-25 among the most severely downregulated. Western blotting further indicated a decrease in Drosha and Dicer proteins which play a key role in miRNA biogenesis, together with an increase of XRN2 known to participate in miRNA degradation pathway. Transcriptome profiling followed by GO and pathway analyses indicated that *A. cinnamomea* induced apoptosis, which was tightly associated with a downregulation of PI3K/AKT and MAPK pathways. Phosphorylation assay further suggested that JNK and c-Jun were closely involved in the apoptotic process. Taken together, our data indicated that the anticancer effect of *A. cinnamomea* can take place within a few hours by targeting multiple proteins and the miRNA system. *A. cinnamomea* indiscriminately induced a global downregulation of miRNAs by simultaneously inhibiting the key enzymes involved in miRNA maturation and activating XRN2 protein involved in miRNA degradation. Collapsing of the miRNA system together with downregulation of cell growth and survival pathways and activation of JNK signaling unleash the extrinsic and intrinsic apoptosis pathways, leading to the cancer cell death.

## Introduction

Hepatocellular carcinoma (HCC) is among the most malignant tumors in humans and known to have highest incidence rate in the developing countries of Southeast Asia and sub-Saharan Africa [1]. Infection with hepatitis type B or C virus, alcoholism and fatty liver disease are found to be the major risk factors associated with HCC tumorigenesis [2]. Recent studies have also identified *Antrodia cinnamomea* (Ac) fungus as a strong anticancer agent, especially against HCC [3], [4].

*Antrodia cinnamomea*, also known as *Antrodia camphorata* or *Taiwanofungus camphoratus*, is a photophobic parasitic fungus naturally growing on the surface the inner cavity of its host plant *Cinnamomum kanehirai*, a bull camphor tree endemic to Taiwan. Over the last few decades, Taiwanese people, especially the aborigines, were using the fruiting body of *A. cinnamomea* to treat a diverse health problems and diseases, including alcohol overconsumption, diarrhea, stomachache, inflammation, and recently against cancer, especially HCC [5]. Its anti-hepatoma potential has been investigated by a number of groups [6], [7], [8], and its ingredient compound antroquinonol is currently on clinical trial (http://clinicaltrials.gov/ct2/show/NCT01134016). A number of *A. cinnamomea* ingredient compounds are known to exert synergistic bioactivities against many types of cancer, either by strengthening the immune system or by directly causing apoptotic cancer cell death: the mycelium of *A. cinnamomea* contains large amount of polysaccharides capable of stimulating the immune system [9]; on the other hand, over 78 compounds were found in the fruiting body and most of those compounds, especially terpenoids which comprise 39 compounds and account for ∼60% of the dry weight of the fruiting body, exhibit profound cytotoxicity against cancer cells [5]. For example, triterpenoids antcin A, antcin C, methyl antcinate A, and 4-acetylantroquinonol B inhibit the proliferation of liver cancer cells [9]. Treatment of human liver cancer cell lines with ethylacetate extract of *A. cinnamomea* fruiting bodies induces apoptosis [8]. Extrinsic and intrinsic cell death pathways are two major pathways in apoptosis. The former is triggered by ligands (e.g. TNF, TRAIL or FasL) which bind to receptors on the cell surface. Then, the oligomerized FADD is recruited to the death-inducing signaling complex (DISC) and binds to caspase-8 and caspase-10 to activate apoptosis. Intrinsic pathway is mediated by members of the BLC-2 family (e.g. BCL-XL, BAD or BAX) resulting in the release of cytochrome c which in turn activates apoptosome through binding of APAF-1 to procaspase-9 [10].

Previous studies on the anticancer effects of *A. cinnamomea* have produced large amount of valuable information. These studies, however, were mainly conducted with prolonged treatment with *A. cinnamomea* ingredient compounds or crude extract. Information regarding the early events is still missing. Here we focused on its early anticancer activities and found that *A. cinnamomea* is able to collapse the microRNA (miRNA) system within the first few hours.

Mature miRNAs are small single-stranded non-coding RNAs of ∼18–24 nucleotides known to post-transcriptionally regulate up to 50% of genes in both plants and animals [11]. Similar to protein-coding genes, miRNA biosynthesis is mediated by RNA polymerase II (Pol II) which transcribes miRNA genes to generate primary miRNAs (pri-miRNAs) which also contain 5′cap and 3′ polyA. Maturation of miRNA transcripts first occur in the nucleus and continue through their subsequent stay in the cytoplasm. In the nucleus, complex of Drosha and DGCR/Pasha cleaves the pri-RNA to produce ∼70 nt hairpin-shaped precursor miRNAs (pre-miRNAs), which are then transported by Exporin-5 to the cytoplasm [12], [13], where the pre-miRNAs are cleaved by the complex of Dicer and TRBL/Loquacious, releasing the double stranded ∼21 bp (miRNA-miRNA* duplex) mature miRNAs. In most cases, the miRNA* strand is degraded, whereas the 5′ end of single-stranded mature miRNA is incorporated into RNA-induced silencing complex (RISC) with Argonaut protein to regulate its target mRNA. Through binding to the 3′UTR of its target gene, a miRNA can either degrade the target mRNA or repress its translation [14]. Compared to mRNAs, the turnover of miRNAs is relatively faster. The level of mature miRNAs is modulated by specific proteins. XRN2 was first reported as an essential factor which specifically degrades single-stranded mature miRNAs in *Caenorhabditis elegans*. miRNAs degrades by XRN2 can modulate functional miRNAs homeostasis in animals [15]. Moreover, XRN2 targeting those miRNAs failed to load into Argonaute and unbound to their target genes for degradation [16].

A number of miRNAs are known to implicate in tumorigenesis. They may function as either tumor suppressor or oncogenes (oncomirs) to regulate cell survival, migration, or apoptosis in various types of cancer, including HCC. miRNA profiling revealed abnormal expression patterns of miRNAs in all tested cancer cells [11], [17], [18]. Although miRNAs play a key role in posttranscriptional regulation and tumorigenesis, to the best of our knowledge, the connection between the anti-cancer effect of *A. cinnamomea* and miRNAs has never been tested or studied.

We took advantage of high throughput next-generation sequencing (NGS), which is a revolutionary technology able to analyze gene expression and regulation at single-nucleotide resolution, to study the effect of *A. cinnamomea* fruiting body extract (AcFBE) against SK-HEP-1(SK) hepatocellular carcinoma cells. By profiling the miRNA and mRNA transcriptomes after 2-hr and 4-hr treatments, we found that AcFBE was able to globally and non-selectively downregulate cancer miRNAs through interacting with multiple miRNA metabolic pathways. This early event represents a novel anticancer mechanism of *A. cinnamomea* fungus that was not known previously and our finding may provoke new therapeutic strategies against cancer.

## Materials and Methods

### Preparation of *A. cinnamomea* fruiting body ethanol extract

Wild type *A. cinnamomea* isolated from a log of *C. kanehirai* was confirmed by the Food Industry Research and Development Institute (FIRDI) of Taiwan using ribosomal ITS (internal transcribed spacer) and LSU (large subunit) rDNA as references. Fruiting bodies of *A. cinnamomea* were harvested from the same log and extracted with absolute ethanol by continuous grinding with mortar and collecting the extract in a test tube. To remove debris, collected extract was centrifuged at 7,000 rpm for 5 min at 4°C, followed by passing through a 0.2-mm filter. To make AcFBE stocks, ethanol in the preparation was subsequently removed by speed vacuum, and the dried AcFBE was dissolved in dimethyl sulfoxide (DMSO) at desired concentrations, split into aliquots and kept at −20°C as stocks. Prior to culture treatment, a calculated amount of stock was mixed with fresh culture medium at room temperature and then used to replace the old culture medium.

### HPLC profiling of AcFBE

For HPLC profiling, AcFBE was dried by rotavapor, re-dissolved in methanol, and passed through a 0.2 µm syringe filter (GHP 13 mm, Acrodisc). 1 µL of the solution (10 µg/µL) was then used for HPLC analysis with Agilent 1200 system. HPLC was performed with Ascentis C18 column (250 mm×4.6 mm, Supelco). Mobile phase: A  =  acetonitrile and B  =  H_2_O. During the first 0–55 min, gradient A was increased from 9.5% to 90% and then stayed at 90% until finished. Flow rate was 0.5 ml/min and the detector wavelength was at 254 and 280 nm.

### Cell culture

Human hepatocellular carcinoma cell line SK-HEP-1 and murine normal liver cell line BNL CL.2 (CL), which was used as control in previous reports [19], [20], were grown at 37°C in Dulbecco's modified Eagle's medium (DMEM) supplemented with 10% of Fetal bovine serum (FBS), penicillin (100 kU/L), streptomycin (100 kU/L), sodium pyruvate (1 mM) and non-essential amino acids (NEAA, 1 mM) in a humidified incubator with 5% CO_2_. The culture medium was changed every other day. 3×10^6^ cells per 10 cm petri dish were seeded prior to treatment with AcFBE.

### MTT cell proliferation/viability assay

It is critical to identify a working concentration of AcFBE which would generate an evident and distinguishable early anticancer effect within hours. To fulfill this purpose, we first treated cells, either SK-HEP-1 liver cancer cell or BNL CL.2 normal liver cell, with variable concentrations of AcFBE for 20 hours. With such excessive duration time, the ideal concentration would be residing in, and should be selected from, those being able to distinguishably impair the viability of cancer cells. Cell viability was measured by using MTT (Sigma), which can be reduced to form purple-colored formazan by mitochondrial dehydrogenase in live cells. The viability rate of the treated cells was compared to that of the untreated counterpart. Duplicates of SK-HEP-1 cells were seeded into 24-well culture plate at 3×10^4^ cells/well and allowed to grow overnight before treatment with AcFBE. After 4 hour or 20 hour of treatment, the medium was removed and cell incubated with MTT working solution for 3 hour. The SK-HEP-1 cells were cultured in the presence of AcFBE at concentrations ranging between 2 mg/ml and 0 mg/ml (untreated control). The absorption was measured with a wavelength of 555 nm using an ELISA reader (Tecan, infinite M200).

### Western blotting

SK-HEP-1 cells were plated at 3×10^6^ cells per 10 cm culture dish 24 hr prior to AcFBE treatment. Cells were then treated with AcFBE at 0.5 mg/ml for 2 hr or 4 hr. After treatment, proteins were extracted with RIPA lysis buffer. 40 µg of cell lysate of each sample was separated by 4–12% gradient NuPAGE (Invitrogen). The primary antibody against Dicer, Drosha, and XRN2 were purchased from Abcam Inc. (Abcam, ab14601, ab12286, and ab72181, respectively). For phosphorylation Analysis, the following antibodies were used: p-c-Fos (Ser32) (Cell Signaling, #5348); c-Fos (Cell Signaling, #2250); p-c-Jun(Ser73) (Cell Signaling, #3270); c-Jun (Epitomics, #1696); p-AKT (Cell Signaling, #4058s); AKT (Cell Signaling, #9272); p-JNK (Cell Signaling, #9251); JNK (Cell Signaling, #9252); p-ERK (Cell Signaling, #4370); ERK1/2 (Cell Signaling, #4695); GAPDH (Epitomics, #2251-1); and Tubulin (Sigma, #T5168). Protein bands were revealed by ECF western blotting kit (Amersham Biosciences) and measured by Typhoon 9400 imager (Amersham Biosciences).

### Construction and sequencing of microRNA and mRNA transcriptome libraries

Total RNA samples were isolated from cultures treated or untreated (DMSO alone) with AcFBE for 2 or 4 hours using Trizol (Invitrogen) following regent manufacturer's instructions.

For miRNA analysis, qualified total RNA samples were applied to miRNA isolation kit (Purelink) to enrich small RNAs, which were subsequently converted into cDNAs. cDNA molecules were then amplified by PCR and purified with MinElute PCR purification Kit (Qiagen). PCR products were displayed by 10% TBE-urea gel (Novex) electrophoresis and fragments of 60–80 bp were excised from the gel, amplified by in-gel PCR, and then eluted using the PCR Micro kit (Purelink). The peparations were then subjected to library construction following sequencer manufacturer's instructions. Next-generation sequencing technology allowed us to study the impact of AcFBE on cancer cells at the sequence level. Libraries of miRNAs isolated from SK-HEP-1 liver cancer cell and BNL CL.2 normal liver cell, each with or without AcFBE treatment (500 µg/ml) for 2 or 4 hours, were first sequenced with SOLiD 3 sequencer. To ensure the reliability, the same experiment was repeated with another batch of SK-HEP-1 cell culture treated with the same experimental conditions, but sequenced by SOLiD 5500xl sequencer.

For mRNA transcriptome analysis, mRNA samples were isolated from total RNA samples and the quality and percentage of RNA samples were evaluated by RNA 6000 Nano kit and Small RNA kit, used in Agilent 2100 bioanalyzer (Agilent Technology). Transcriptome libraries were then constructed by following a protocol modified from that developed by Tang et al. for single cell transcriptome analysis [21]. In short, cDNA libraries were generated from mRNA samples by reverse transcription, amplified by standard PCR, sonicated into small fragments and ligated to adaptors. Libraries then amplified onto beads by emulsion PCR. Followed by 3′ end modification, and then deposition onto flowchip for sequencing by SOLiD3 sequencer.

### miRNA and mRNA data processing and analysis

miRNA and mRNA libraries of both AcFBE treated and untreated were sequenced at two different time points (2 hr and 4 hr). Preprocessing of raw data generated by NGS follows a standard procedure which identifies qualified sequence reads for downstream analysis. For each library of miRNA and mRNA, the qualified sequence reads also had to pass phred quality score (QV≥20 equivalent to 99% accuracy. To process four libraries (2 hr or 4 hr for each treated or untreated) of sequences with length 35 bp generated from SOLiD3 or SOLiD 5500xl machine, we built an in-house pipeline using shell script combined with custom made perl script. To avoid adapter trimming process using duplicated dataset, identical sequences were clustered and assigned a unique tag with count (e.g tag_100). Cutadapt [22] was used to trim ligated adapter at most 2 mismatches. Then, cleaning polyN and removing identical reads shorter than 16 and longer than 30 were processed to match the range adopted by miRBase using custom made perl script.

To identify known and novel miRNAs, we eliminated tRNA and rRNA sequences from the libraries by mapping sequence reads against Rfam (http://rfam.sanger.ac.uk/) and tRNA (http://lowelab.ucsc.edu/GtRNAdb/) using Bowtie program [23]. Sequence reads mapped to those databases with perfect match were removed from the libraries. In addition, repetitive sequences in that mapped to Repbase (http://www.girinst.org/repbase/) were also removed. Then, miRBase (http://www.mirbase.org/) was employed to identify the known miRNAs. The remaining sequence reads were mapped to UCSC hg19 database and only sequence reads successfully mapped to UCSC hg19 allowing two mismatches were considered as novel miRNAs. In our miRNA profiling, we only used known miRNAs, mapped by at least 2 reads for analysis.

Transcriptomes generated from mRNA of SK-HEP-1 cells treated or untreated with AcFBE were sequenced with SOLiD 3 and profiled to further understand the anticancer effects of AcFBE for both mRNA and miRNA transcriptome analyses, the outlier was identified by linear regression. We obtained more significant p-value by paired Student's t-test and higher correlation coefficient value in comparison with entire data for analysis to remove noise expression data in the cross-library.

The miRNA and mRNA expression data have been deposited to the Gene Expression Omnibus (GEO, http://www.ncbi.nlm.nih.gov/geo/) with accession number GSE48327.

### Scatter plotting and box plotting of miRNA data

Both boxplots and scatterplots were produced with R language. To improve the clarity of data presentation, we removed entries with zero value and converted values to log_2_ scale. Boxplots were generated by built-in boxplot() function, while scatterplots were generated by geom_point() function in the package of ggplot2.

In both transcriptome and miRNA analyses, the outlier is identified by linear regression. By removing the outliers in the cross-library experiments, we obtain more significant p-value by paired Student's t-test and higher correlation coefficient value in comparison with using entire data for analysis.

## Results and Discussion

### Selection of AcFBE concentration and the duration time for treatment

MTT assay showed 15% and 46% survival rates, respectively, for SK-HEP-1and BNL CL.2 cells with treated 500 µg/ml AcFBE for 20 hr. Results also showed that the viability of the SK-HEP-1 cells was impaired in a dose-dependent manner, and at concentrations equal to or greater than 500 µg/ml ( = 0.5 mg/ml) over 90% of SK-HEP-1 showed sign of apoptosis. We also found that at the concentration of 500 µg/ml about 50% of SK-HEP-1 cells were affected within 4 hours, and the apoptotic effect increased along with concentration. Based on the results, 500 µg/ml was selected as the working concentration. To reinforce the reliability, experiments were performed for 2- and 4-hour time points.

### HPLC profile of AcFBE

Constituents of the fruiting body of *A. cinnamomea* was previously analyzed with HPLC by Geethangili et al [5]. In their report, over 78 compounds were identified. Although the constituents are expected to vary among samples, a detailed analysis with HPLC for every sample is not necessary. As such, we decided to just take a quick glimpse of our AcFBE sample with HPLC using antrocamphin A as a reference. Location of antrocamphin A is cross referenced from another HPLC experiment run sequentially with the AcFBE column (**Figure S1**).

### Workflow

**Figure 1:**
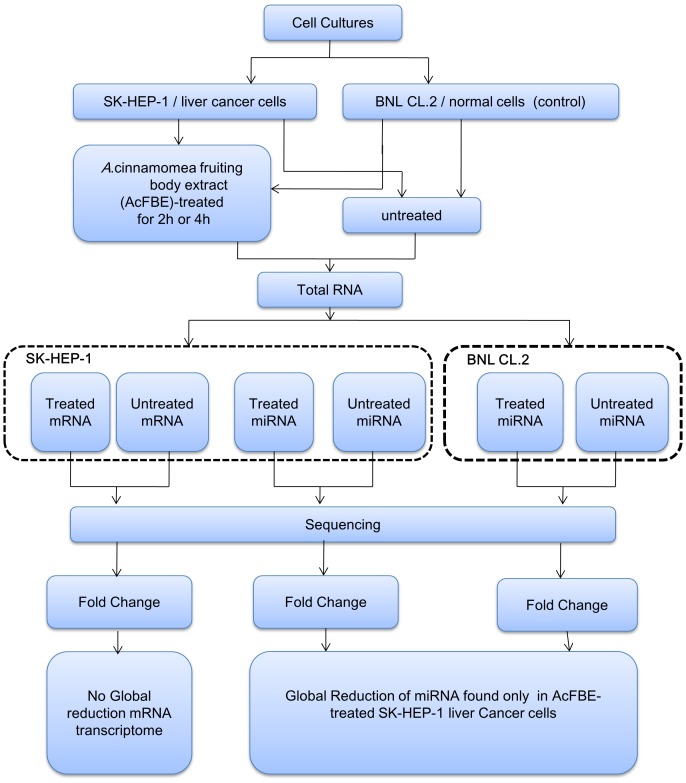
MicroRNA analysis workflow. Both experimental and control cells were treated, or untreated (control, DMSO alone was added), with 500 µg/ml AcFBE (experimental) for 2 or 4 hours. MicroRNAs were extracted, sequenced, and compared. Both cross-time and cross-experiment analyses were performed to test the reliability and AcFBE effect, respectively. The same experiments were repeated once to ensure reliability. Messenger RNA transcriptomes were also prepared for further understanding of the nature of miRNA fluctuation.

For systematic study of the perturbation of miRNA and mRNA transcriptomes resulted from AcFBE treatment, a workflow was designed (**Figure 1**). Cultures of SK-Hep-1 liver cancer cells or BNL CL.2 mouse primary live cells (control) were treated or untreated with AcFBE for 2 or 4 hrs. Total RNA was isolated from each culture and used for miRNA or mRNA sequencing and analyses.

### MicroRNA analysis

A number of known miRNAs, ranging between 228 to 319 miRNAs per library, were detected among these small RNA libraries. The library statistics are shown on **Table S1** and **S2**.

### Cross-time comparison revealed a linear correlation for both untreated and AcFBE-treated and untreated samples

**Figure 2:**
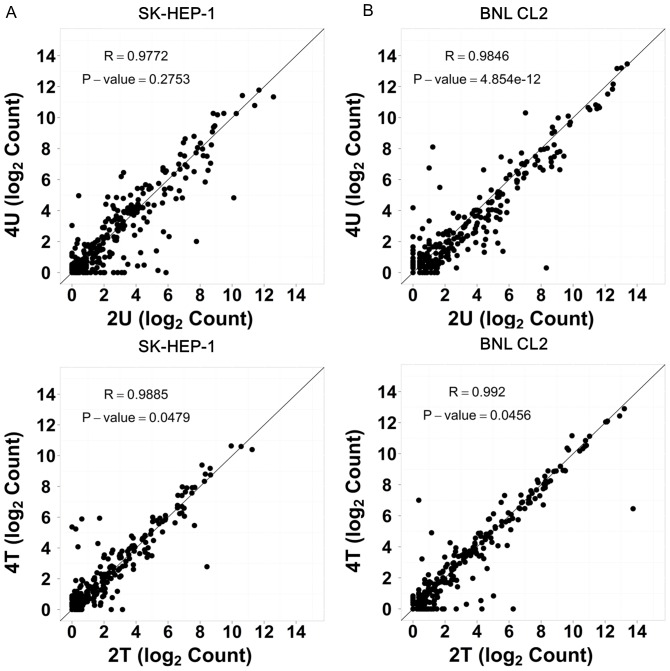
Cross-time point comparison of miRNA expression by scatter plotting. SK-HEP-1 miRNAs (**A**) and BNL CL.2 (control) miRNAs (**B**) both sequenced by 5500xl and analyzed with scatter plotting to test the consistency of data extrapolation from 2 hr time point to 4 hr time point. The correlation coefficient (R) and P-value of each library were calculated using Student's t-test. A linear correlation was observed in all cases.

To test the reliability of miRNA data, we did a cross-time comparison by plotting 2U (untreated for 2 hr) dataset against 4U (untreated for 4 hr) dataset and 2T (treated for 2 hr) dataset against 4T (treated for 4 hr) dataset. This was conducted for both SK-HEP-1 liver cancer cell and BNL CL.2 control cell. Results all showed a linear correlation between 2 hr and 4 hr samples, indicating that these miRNA datasets are highly reliable (**Figure 2**).

### Cross-experiment comparison indicated a global reduction of cancer miRNAs by AcFBE

**Figure 3:**
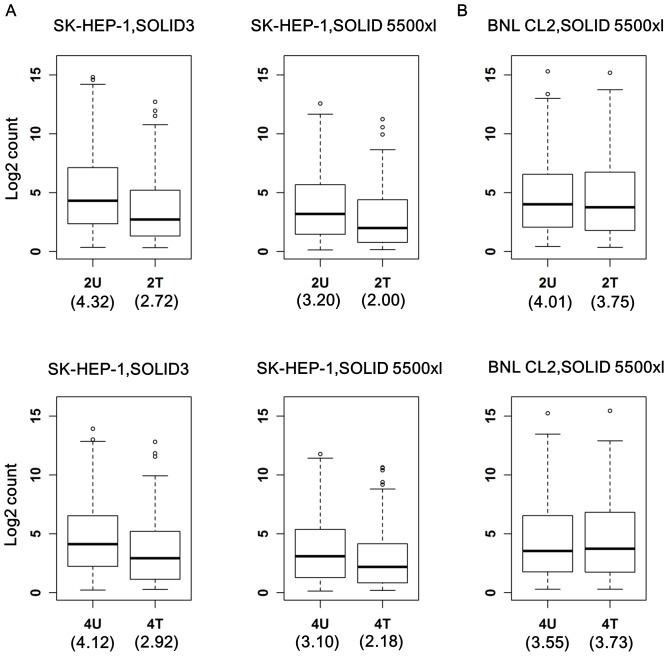
Boxplots showing cross-experiment comparison on miRNA expression between AcFBE-treated and –untreated samples. The distributions of miRNA expression level (inner boxes) and medians (horizontal line in each inner box, value shown in the parentheses right below the outer box) are presented by box plotting for each (untreated vs. treated) pair for each time point (either 2 hr and 4 hr time point and for both SK-HEP-1 and BNL CL.2 normal cells. Experiments of SK-HEP-1 cancer cells were repeated and the duplicates were sequenced separately by two types of sequencers (shown above). (**A**) SOLiD 3 miRNA dataset produced from SK-HEP-1 and SOLiD 5500 miRNA dataset produced from another batch of SK-HEP-1. (**B**) miRNA dataset produced from BNL CL.2 normal cells (control).

We then conducted a cross-experimental condition comparison between the AcFBE-treated and untreated samples. Surprisingly, we observed a dramatic decrease in the level of most miRNAs in SK-HEP-1 cancer cell in response to AcFBE treatment (**Figure 3**). Furthermore, this phenomenon was found only in SK-HEP-1 cancer cell, not the AcFBE-treated BNL CL.2 control. For SK-HEP-1 cell, the log_2_ count of (untreated vs. treated) median pair were (4.32 vs. 2.72) and (3.20 vs. 2.00) for 2 hr duplicates, and (4.12 vs. 2.92) and (3.10 vs. 2.18) for 4 hr duplicates (**Figure 3A**). Both duplicates showed the same trend of miRNA reduction. In contrast, the (untreated vs. treated) median pair of BNL CL.2 cells were (4.01 vs. 3.75) for 2 hr samples and (3.55 vs. 3.73) for 4 hr samples showing no evident reduction in response to AcFBE treatment (**Figure 3B**).

### Most cancer miRNAs in AcFBE-treated SK-HEP-1 cells were downregulated by at least two folds

**Figure 4:**
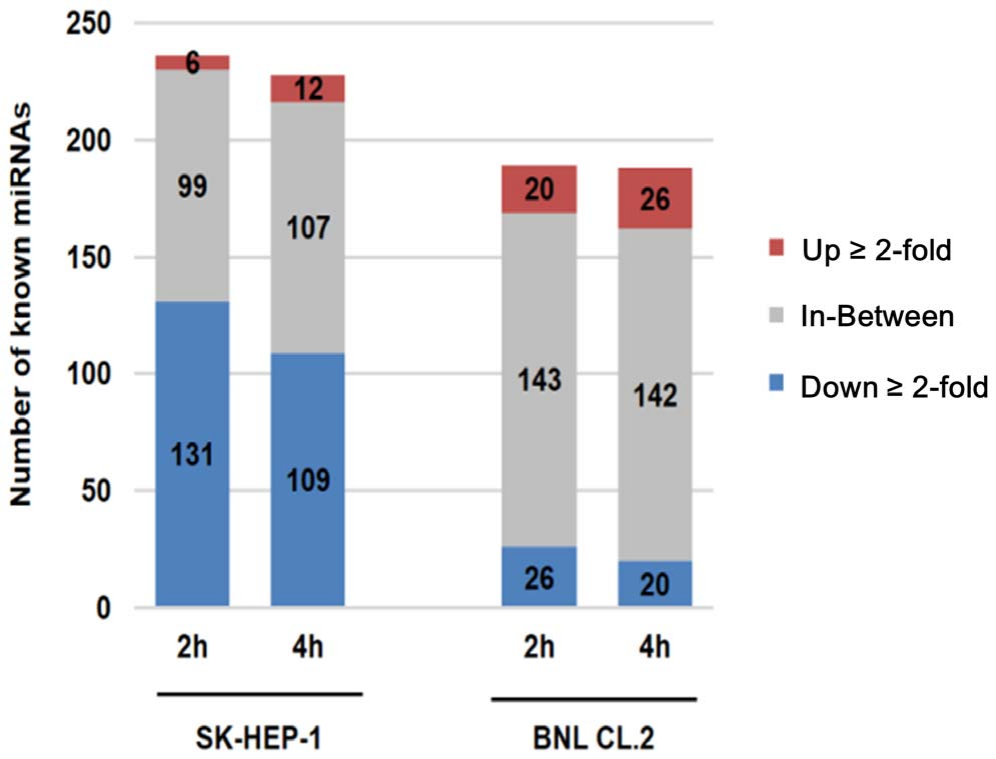
Expression profiles of miRNAs for SK-HEP-1 and BNL CL.2 by treated AcFBE. To compared the expression level of known miRNAs affected by treated AcFBE for SK-HEP-1 and BNL CL.2 cells at 2 hr and 4 hr.

To further understand the nature of miRNA downregulation, we analyzed known miRNAs that satisfied the following criteria: 1) count ≥10 in either treated or untreated library or both, and 2) up or down regulated by at least 2-fold (treated compared against untreated). Expression of known miRNAs demonstrated 55.5% (131/236) and 47.8% (109/228) of known miRNAs found downregulated in SK-HEP-1 at 2 hr and 4 hr respectively, but only 13.8% (26/189) and 10.6% (20/188) of known miRNAs downregulated in BNL CL.2 cells at 2 hr and 4 hr (**Figure 4**).

In contrast, the untreated sample and the normal liver cells remained unaffected. Even more surprisingly, the effect took place within 2 hours. Acting as regulators of mRNAs, miRNAs seem to be much more sensitive than mRNAs to the ingredient compounds of *A. cinnamomea* fruiting bodies. As such, a large part of the anticancer effect of *A. cinnamomea* seemed to be mediated by miRNAs and such miRNA-mediated anticancer effect seemed to be resulted from a “blind shooting” mechanism which does not discriminate or in favor of particular types of miRNAs.

### Both oncogene clusters and individual oncomirs were downregulated

The expression of oncomirs in major oncogenic clusters miR-17-92 and miR-106b-25 together with oncomirs miR-21, miR-191 are among the most severely impaired. In 2 and 4 hours AcFBE-treated SK-HEP-1 cells, 70% of oncomirs at least 50% lower than the AcFBE-untreated SK-HEP-1 cells. The target mRNAs of these oncomirs are encoded by pro-apoptotic genes*PTEN*, *p21*, *BIM* and *Caspase-3/7* [10], [24]. Inhibition of these pro-apopotic genes by the oncomirs clusters results in cell proliferation due to the loss of function in apoptosis. *PTEN* (phosphatase and tensin homologue) gene is known as a tumor suppressor and often found mutated in cancer. Gene *p21* is a downstream target of *p53* and acts as regulator of cell cycle progression at G1. BH3-only protein *BIM* functions as an activator to *BAX* [10]. Our results suggest that AcFBE targets on the oncomir clusters which would otherwise repress pro-apoptotic genes to disrupt the apoptosis mechanism (**Table 1**).

**Table 1:** Expression of miRNAs encoded by miR-17-92 and miR-106b-25 oncogenic clusters.

miRNAs	MiRNA expression (RPKM)			
	2U	2T	4U	4T
**miR-17** #	482.5	134.2	710.3	198.8
**miR-18a** #	0.3	2.4	30.2	0.2
**miR-19a** #	133.0	142.8	396.7	239.0
**miR-20a** #	195.6	2.3	442.2	60.3
**miR-19b** #	541.5	394.3	1163.1	577.8
**miR-92a** #	258.7	178.3	326.7	189.3
**miR-106b** *	11.2	4.0	16.2	4.3
**miR-93** *	217.3	22.2	3.0	13.7
**miR-25** *	292.9	115.6	186.0	102.7

(#) miR-17-92 cluster; (*) miR-106b-25 cluster; 2U, 2-hr untreated; 2T, 2-hr treated; 4U, 4-hr untreated; 4T, 4-hr treated.

### Most of the top 20 most downregulated miRNAs identified from samples treated for 2 hours remained downregulated after two more hour treatment

**Figure 5:**
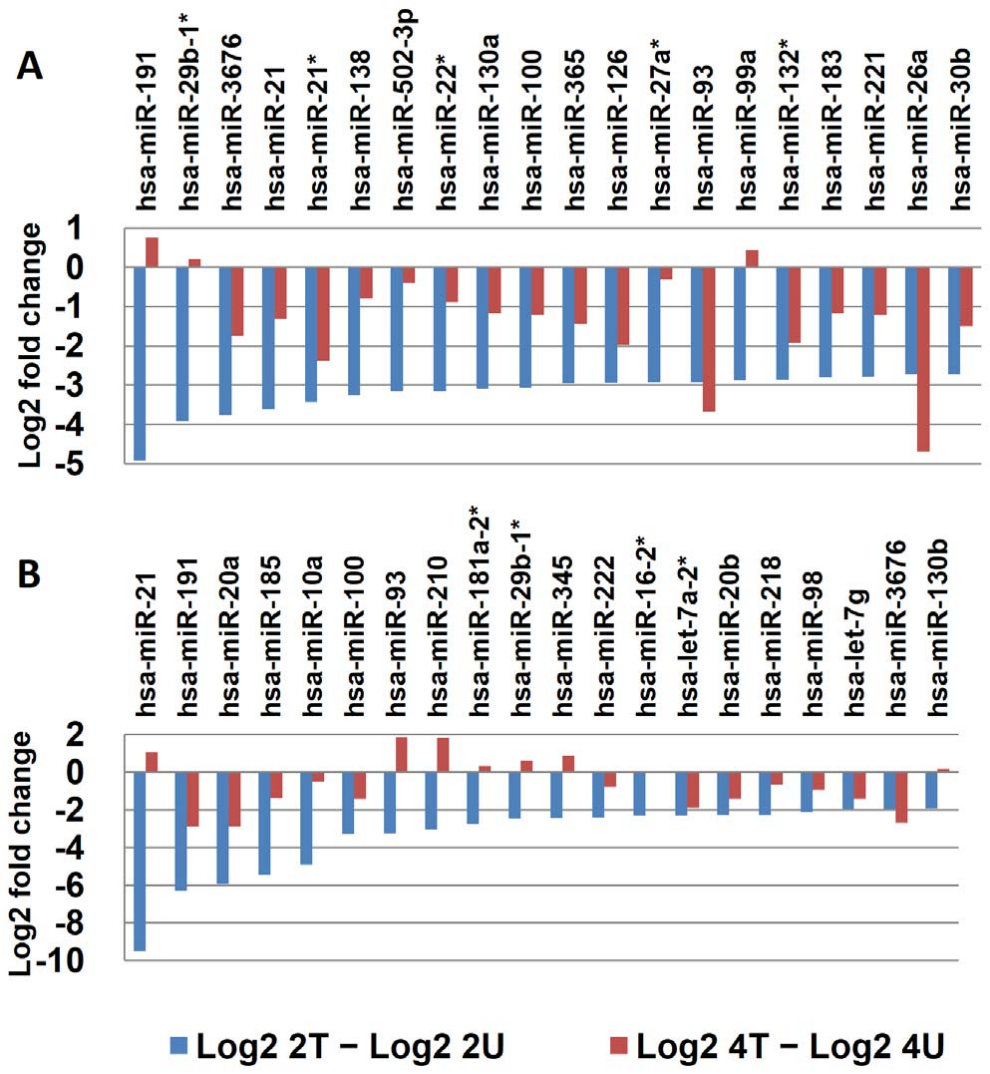
Cross-time comparison for the top 20 most downregulated miRNAs identified from SK-HEP-1 cells treated with AcFBE for 2 hours. (A) First batch of quality miRNAs (sequenced by SOLiD 3) from 2 hr AcFBE-treated or –untreated SK-HEP-1 cells were normalized and used to calculate log_2_ fold changes. Values of the untreated were subtracted from the treated. The top 20 most significantly downregulated miRNAs were identified and compared with their levels at 4 hr time point. (B)Same as A, but prepared from another batch of SK-HEP-1 cells (repeated samples sequenced by SOLiD 5500xl).

To analyze the trend of miRNA downregulation, we selected the top 20 most significantly downregulated miRNAs from the 2 hr time point samples (treated vs. untreated) and compared with their corresponding values from the 4 hr time point samples. Results from duplicates, independently sequenced by SOLiD 3 (**Figure 5A**) and SOLiD 5500xl (**Figure 5B**), are both shown for comparison. Although the results showed a strong correlation at 2 hr time point among those most significantly downregulated miRNAs (e.g. the top 4 all showed up in both duplicates), but the correlation seemed to gradually disappear with time, following a random pattern.

### All the top 10 most upregulated miRNAs at 2-hr time point became downregulated after two more hours of treatment

**Figure 6:**
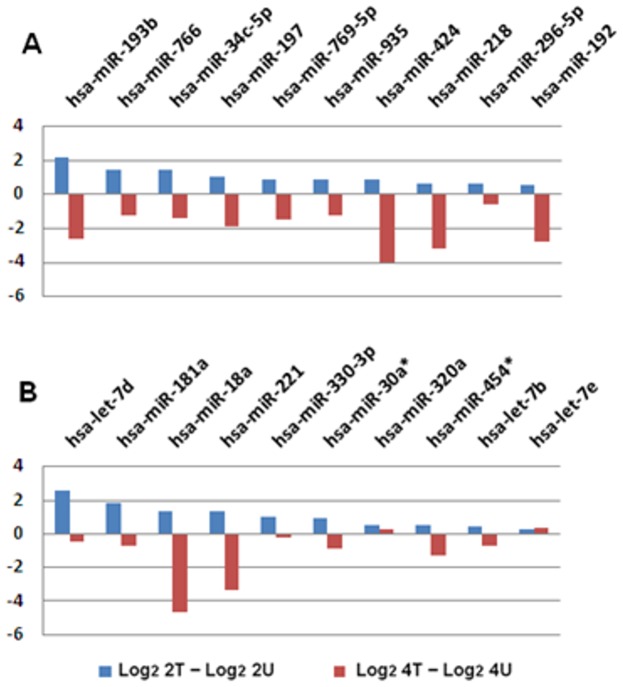
Cross-time Comparison (2 hr vs. 4 hr) of the top 10 most significantly upregulated miRNAs. (**A**) First batch of quality miRNAs (sequenced with SOLiD 3) from 2 hr AcFBE treated or untreated SK-HEP-1 cells were normalized by (actual reads/quality reads) ×1,000,000 and used to calculate the log_2_ fold change. Values of the untreated were subtracted from the treated followed by a sorting to identify the top 10 miRNAs with highest positive values which were then compared with their levels at 4-h time point. (**B**) Same as A, but prepared from another batch of SK-HEP-1 cells (repeated samples sequenced with SOLiD 5500xl).

Among the miRNAs that showed positive response to AcFBE treatment at 2 hr time point became downregulated at 4 hr time point, but without evident correlation (**Figure 6**).

It is noteworthy that potent oncomirs (oncogenes), hsa-miR-191 (miR-191) and hsa-miR-21 (miR-21), are known to be upregulated and implicated in MCF-7 breast cancer and hepatocellular carcinoma tumorigenesis. Inhibition of hsa-miR-191 by 2-O-metoxyethyl (MOE) anti-miRNA oligonucleotide reduced proliferation and increased apoptotic response in dose-dependent manner [24]. Similarly, inhibition of miR-21 by transfecting MCF-7 cells with anti-miR-21 oligonucleotides resulted in decreased cell proliferation and a BCL-2-mediated intrinsic apoptosis [25]. Here, we found that AcFBE exhibited strong inhibition on both miR-191and miR-21 oncomirs in SK-HEP-1 cells within two hours. The global inhibition of oncogenic miRNAs, including miR-21 and miR-191, together with oncomirs encoded by miR-17-92 and miR-106b-25 oncogenic clusters, which mainly target on pro-apoptotic genes [10], may be the major causes leading to acute cancer cell death which occurred within two hours.

### Western blotting indicated that both miRNA biogenesis pathway and miRNA degradation pathway were targeted by AcFBE

**Figure 7:**
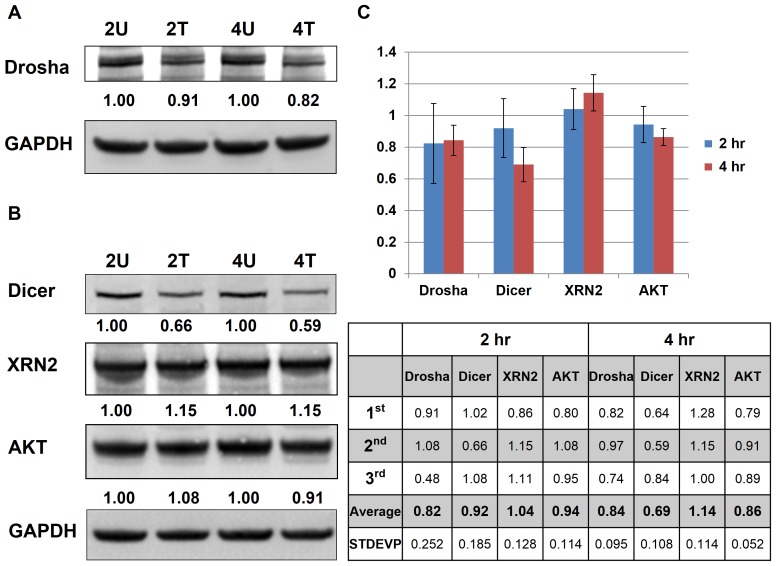
Western blot analysis of SK-HEP-1 samples with or without AcFBE treatment. Western blots showed a decrease in Drosha (**A**) and Dicer (**B**) proteins which are key enzymes of the miRNA biogenesis pathway. This is accompanied by a minor increase in XRN2 protein, involved in miRNA degradation. A minor decrease of AKT (involved in PI3K pathway) was also observed. Cancer cell line, SK-HEP1 hepatocarcinoma cell; Drug, *A. cinnamomea* fruiting body extract (AcFBE); 2U, sample treated with DMSO for 2 hr; 2T, sample treated with AcFBE at 0.5 mg/ml for 2 hr; 4U, sample treated with DMSO for 4 hr; 4T, sample treated with AcFBE at 0.5 mg/ml for 4 hr. (**C**) Summary bar chart of triplicate western blot experiments. 2 hr, samples treated with AcFBE for 2 hours; 4 hr, samples treated with AcFBE for 4 hours. Protein levels relative to untreated controls are shown blow.

We employed Western blotting to investigate the AcFBE effects on miRNAs biogenesis and degradation pathways. Both Drosha and Dicer are the key enzymes of miRNA biogenesis, and XRN2 has been reported as an important enzyme for miRNA degradation. Western blotting indicated that the protein levels of Drosha and Dicer, key enzymes for miRNA biogenesis, were both negatively regulated by AcFBE (**Figure 7A, B**). In contrast, XRN2 protein, which is known to degrade miRNAs, was found to be positively regulated by the same treatment. Upregulation of XRN2 was accompanied by a 1.8-fold increase in mRNA level. These phenomena were confirmed by triplicate western blot experiments (**Figure 7C**). Since Drosha and Dicer regulate miRNAs without specificity or discrimination, this result also supports the blind shooting mechanism.

### Characterization of Genes and Functional Annotation

The next question is how the global downregulation of miRNA is associated with cancer cell death. Apoptosis regulation by miRNA has been intensively reviewed [15], [36], and it is well established that cancer miRNA network play a key role not only in tumorigenesis, but also in further cancer development. In theory, the collapse of miRNA network alone would inevitably lead to cancer apoptosis. However, further understanding of the molecular mechanisms underlying this process would rely on the study of mRNA transcriptomes. To answer these questions, we analyzed the mRNA transcriptome of the SK-HEP-1 cells treated with AcFBE. The transcriptome library statistic is shown in **Table 2**.

**Table 2:** Transcriptome library statistics.

Description	Libraries			
	2U	2T	4U	4T
**Original Sequencer output**	25,501,838	16,941,503	17,394,007	19,225,986
**Total Qualified Reads (%)**	8,407,306 (32.97)	9,260,296 (54.66)	8,061,838 (46.35)	9,573,485 (49.79)
**Unique reads (uReads)**	1,548,122	1,702,518	1,561,795	1,695,814
**Total-to-unique (T2U) ratio**	5.43	5.44	5.16	5.65
**Total Count of Mapped uReads**	6,739,968	7,386,787	6,507,959	7,578,096
**Mappable uReads**	1,275,397	1,468,620	1,343,608	1,418,521
**Number of genes**	9,377	10,080	9,550	9,773

2U, 2-hr untreated; 2T, 2-hr treated; 4U, 4-hr untreated; 4T, 4-hr treated.

**Figure 8:**
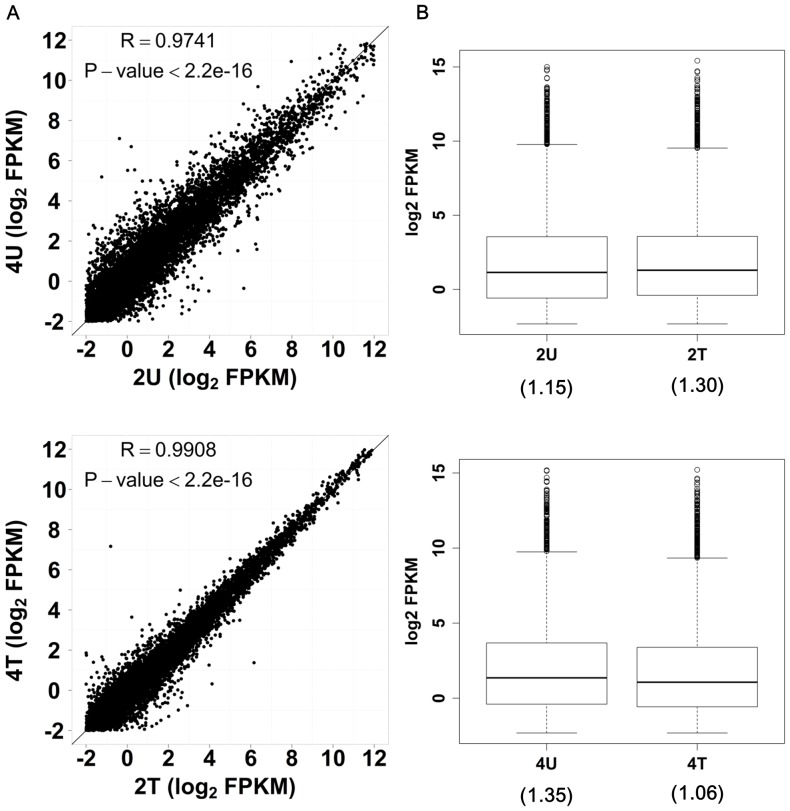
Transcriptome analyses of SK-HEP-1 liver cancer cells treated or untreated with AcFBE. All transcriptomes were analyzed with RNA-Seq approach. The expression level of an mRNA is represented as FPKM (fragments per kilo base of exon per million fragments mapped). (**A**) Scatter plots reveal a linear correlation in mRNA expression between AcFBE-untreated SK-HEP-1 samples (2U vs. 4U) and also between AcFBE-treated SK-HEP-1 samples (2T vs. 4T). The correlation coefficient (R) and P-value of each library were calculated using Student's t-test. The profile of treated sample seems to be less scattered than the untreated sample.(**B**) Box plots show the expression distribution and median (inside parenthesis) for each transcriptome library.

In our sequencing data, we extracted over 8 million quality reads from each mRNA transcriptome libraries. These quality reads were mapped against UCSC hg19 database with Bowtie and Cufflinks [26] by allowing 2 mismatches. Scatterplots showed a linear correlation in mRNA expression between the untreated samples (2U vs. 4U), and between the treated samples (2T vs. 4T) (**Figure 8A**). Noticeably, no global downregulation was detected in AcFBE-treated mRNA samples (**Figure 8B**). That is, SK-HEP-1 mRNA transcriptome did not show an instant and dramatic AcFBE shock as its miRNA transcriptome did, although a small decline in median seemed to appear in the 4 hr AcFBE-treated sample.

### Gene Ontology analysis

**Figure 9:**
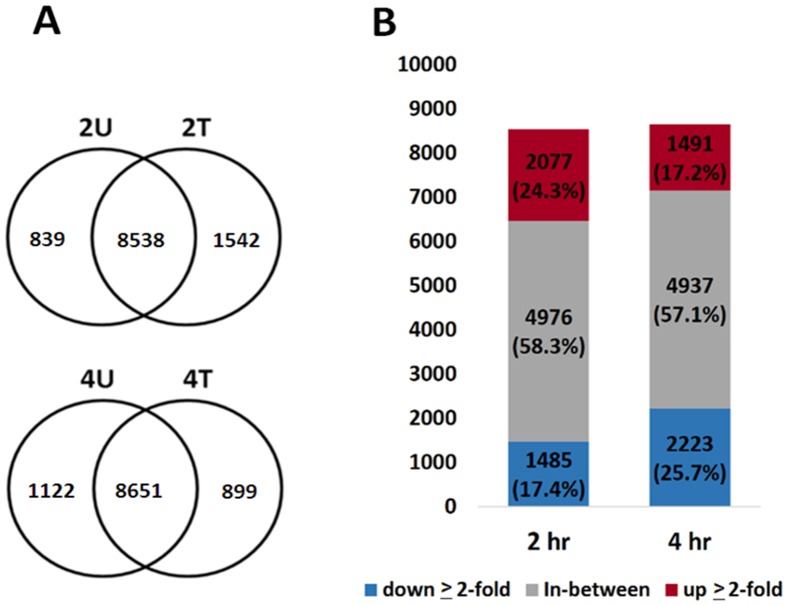
The overlapping genes in transcriptome libraries. (**A**) Overlapping gene expression between the AcFBE-treated and –untreated SK-HEP-1 cancer cells in transcriptome libraries. This diagram shows the numbers of overlapped genes between the AcFBE-treated and untreated SK-HEP-1 cells. (**B**) Bar chart showing the number of responsive genes after treated with 500 µg/ml AcFBE for 2 or 4 hours. 2T, 2 hr treated; 2U, 2 hr untreated; 4T, 4 hr treated; 4U, 4 hr untreated. Up-expressed label red color and down-expressed label blue color.

The numbers of identified protein-coding genes per library ranged between 9,377 and 10,080. Numbers of overlapped genes between the treated and untreated samples are shown in (**Figure 9A**). Among these genes, 41.7% (3,562 genes) and 42.9% (3,714 genes) were found to respond to AcFBE treatment (with fold change ≥ 2 or ≤−2) for 2 hr and 4 hr time points, respectively. Overall, the percentages of mRNA transcripts downregulated were 17.4% (1485/8538) and 25.7% (2223/8651), respectively, at 2 hr and 4 hr (**Figure 9B**). The globally downregulation in miRNA transcriptome was not found in mRNA transcriptome.

GO term analysis indicated that AcFBE targeted apoptosis associated genes. We selected genes from our mRNA transcriptome data which passed our fold change criteria (fold change ≥ 3 or ≤−3 both in 2 hr and 4 hr) for Gene Ontology (GO) analysis using public tool DAVID for functional annotation with fisher-exact test. Interestingly, the most significant GO terms of Biological process (BP) were apoptosis related. Also, the significant terms on Molecular Function (MF) revealed that selected genes involved heavily in transcription (**Table 3**). We reasoned that mRNA expression of AcFBE treated SK-HEP-1 cells at early apoptosis involving both pro-apoptotic and anti-apoptotic functional genes in transitional phase.

**Table 3:** GO analysis.

GO Term	Term	p-value	Benjamini
Biological Process (BP)	Regulation of apoptosis	1.3E-7	2.3E-4
	Regulation of programmed cell death	1.7E-7	1.5E-4
	Regulation of cell death	1.8E-7	1.1E-4
Molecular Function (MF)	Transcription factor binding	4.8E-6	2.1E-3
	Transcription cofactor activity	2.7E-4	5.9E-2
	Transcription activator activity	3.5E-4	5.1E-2
Cellular Component (CC)	Nuclear lumen	2.2E-11	6.9E-9

### Analysis of apoptosis-associated genes and pathways

**Figure 10:**
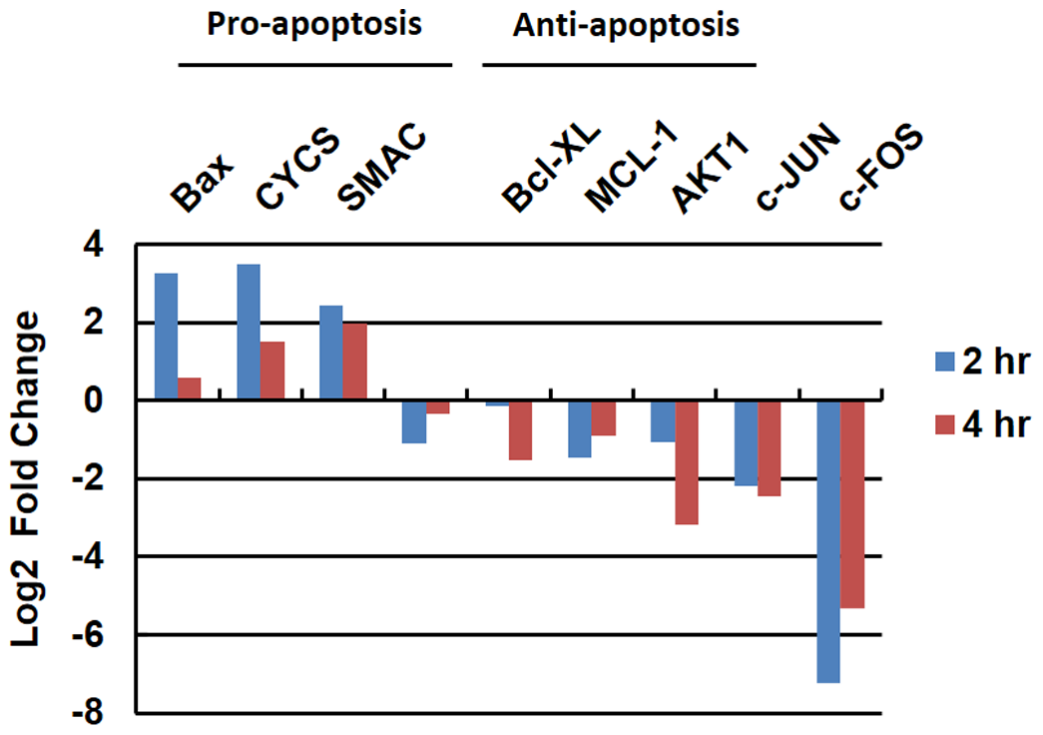
Alterations in the expression of pro-apoptotic and anti-apoptotic genes. SK-HEP-1 liver cancer cells were treated (or untreated) with *A. cinnamomea* fruiting body extract for 2 or 4 hours. Transcriptomes from treated samples were analyzed and compared against the untreated counterparts to reveal the alteration in the expression levels of pro- and anti-apoptotic genes.

We further analyzed mRNA transcriptome data to study the AcFBE effect on biological pathways and identified alterations in expression level of certain apoptosis associated genes in response to AcFBE treatment. AcFBE increased the level of pro-apoptosis mRNAs encoded by *CYCS* (cytochrome c), *DIABLO* (SMAC) and *BAX* and decreased the level of anti-apoptotic miRNAs of *NFκB, BCL-XL*, *MCL-1* and *AKT1* (**Figure 10**).

**Figure 11:**
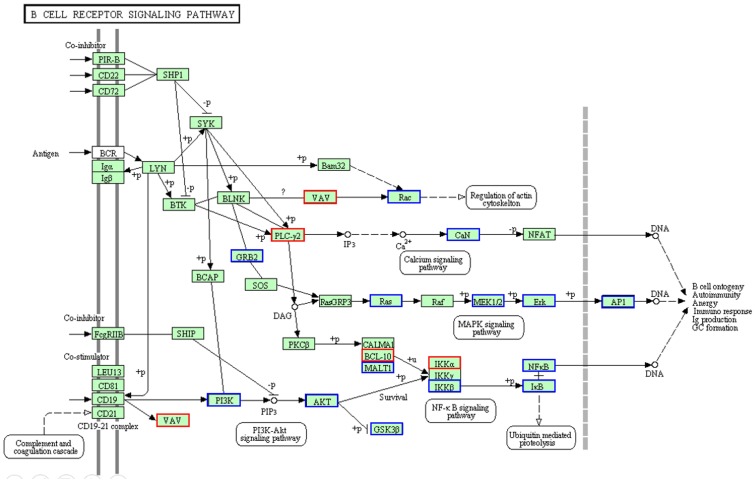
AcFBE-induced downregulation of MAPK and PI3K/AKT pathways as demonstrated by B cell signaling network using KEGG diagram. Transcriptomes generated from messenger RNAs of SK-HEP-1 liver cancer cells with or without AcFBE treatment for 2 hours were analyzed and plugged into KEGG database for pathway analysis. Through the process, PI3K/AKT/IKKβ/NFκB and MAPK were identified among the most significantly affected pathways. Upregulated pathway genes are highlighted in red boxes, while downregulated pathway genes highlighted in blue boxes.

We used DAVID and KEGG pathway database to study pathway fluctuation and found that MAPK and PI3K/AKT signaling pathways were among the most significantly downregulated pathways by AcFBE. These pathways are displayed in B cell immunological functions pathway in KEGG database. As shown in **Figure 11**, *RAS, MEK, ERK, c-Fos* and c-*Jun* genes in MAPK signaling pathway together with *PI3K, AKT, IKKβ*, and *NFκB* genes in PI3K/AKT pathway were all transcriptionally inhibited by AcFBE.

The activation of MAPK and PI3K/AKT pathway is fundamentally important for cell proliferation and survival. Negative regulation of PI3K/AKT and MAPK/ERK pathway enhances apoptosis and causes cell cycle arrest [27], [28], and PI3K/AKT pathway activates apoptosis by withdrawing survival signal c-Myc [29]. AcFBE inhibition of PI3K/AKT/NFKB survivin signaling could lead to increased apoptosis [30].

### Phosphorylation analysis

**Figure 12:**
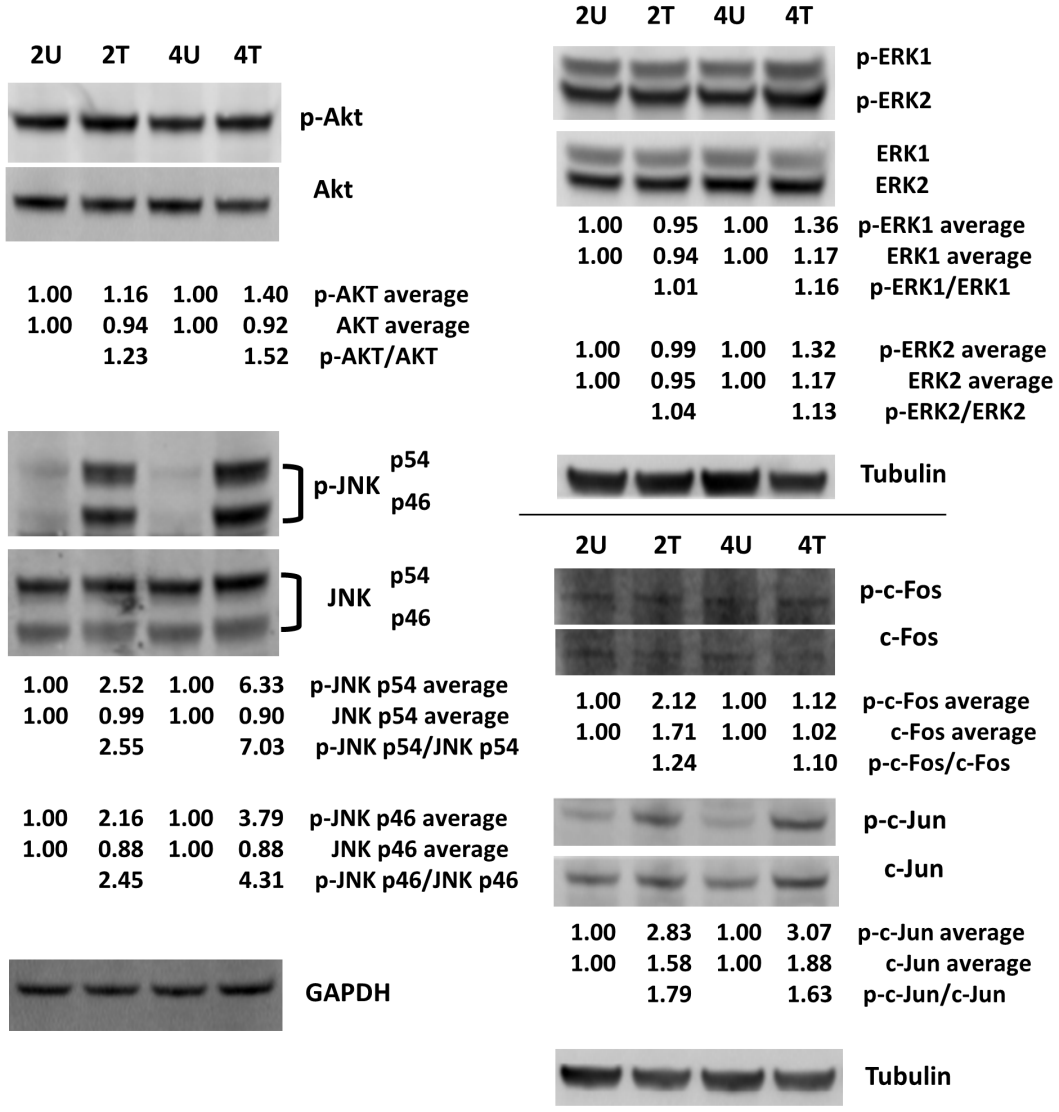
Western blot analysis of the phosphorylated and unphosphorylated forms of AKT, ERK, JNK, c-Fos, and c-Jun. SK-HEP1 liver cancer cells were treated with (at 0.5 mg/ml) or without AcFBE for 2 or 4 hrs. Immunoblotting experiments were repeated twice using GAPDH or Tubulin as controls. The duplicates were averaged and shown below each protein band, and the phosphorylated-to-unphosphorylated ratios were then calculated and shown below the treated samples. Protein bands were from the first set of immunoblotting experiment. The normalized protein level of untreated samples was set as 1.00 for comparing to their corresponding AcFBE-treated samples. 2U, untreated for 2 hrs; 2T, treated for 2 hrs; 4U, untreated for 4 hrs; 4T, treated for 4 hrs.

To further understand the roles of PI3K/AKT and MAPK pathways in AcFBE-induced apoptosis, we conducted phosphorylation analysis on AKT and MAPK/ERK, together with some key components of these pathways in AcFBE-treated SK-HEP-1 liver cancer cell. Results showed no significant alterations in phosphorylated-to-unphosphorylated ratio for ERK1, ERK2, and c-Fos (**Figure 12**). However, the phosphorylated-to-unphosphorylated ratio of JNK/SAPK (c-Jun N-terminal kinase, also known as stress-activated protein kinase) was elevated up to 4.3-fold and 7.0-fold for JNK p46 and JNK p54, respectively, by AcFBE within 4 hours, while the same phosphorylation ratio of c-Jun and AKT increased by 1.6-fold and 1.5-fold, respectively. A number of reports have implicated the role of JNK/SAPK in apoptosis, either induced by serum starvation, growth factor withdrawal, or carcinogen [31], [32], [33]. Reports have also shown that activation of JNK/SAPK would in turn phosphorylate c-Jun, and the phosphorylated c-Jun may be necessary for the downstream events of apoptosis [31], [34]. In addition to the globally negative effect of AcFBE on the miRNA system, these results indicate the presence of independent anticancer mechanisms of AcFBE on transcription (of nascent mRNAs) and phosphorylation (of existing proteins). Results also suggest that both JNK and c-Jun may play important roles in AcFBE-induced apoptosis.

## Conclusion

Here, we employed deep sequencing followed by miRNA and mRNA profiling to study the early anticancer events of *A. cinnamomea*. Using this novel strategy, we demonstrate that the global downregulation of miRNAs represents an early anticancer mechanism of *A. cinnamomea*, which occur within hours and such global downregulation of miRNAs did not occur to normal liver cells.

We also provide strong evidence showing that the global repression of cancer miRNAs is mediated by inhibition of miRNA biogenesis pathway, as well as by activation of miRNA degradation pathway. Thus, *A. cinnamomea* exerts its early inhibitory effect upon cancer miRNA system by disrupting multiple miRNA metabolic pathways, causing indiscriminative reduction of miRNA species. Such broad perturbation of the miRNA regulatory system inevitably leads to cancer cell death.

Gene Ontology analysis on mRNA transcriptomes of *A. cinnamomea*-treated hepatocarcinoma cells associated the fluctuation of gene expression with apoptosis. Both MAPK and PI3K/AKT pathways showed sign of weakening in transcriptional potential within hours. In parallel, phosphorylation of both JNK and c-Jun increased significantly, suggesting that *A. cinnamomea*-induced hepatocarcinoma cell apoptosis may be mediated by JNK signaling as well.

Our results are consistent with reports showing that downregulation of PI3K/AKT and MAPK signaling pathways is well associated with apoptosis. For example, in their study of the role played by occludin (an integral membrane protein in normal cells) in tight junction expression and apoptosis in hepatocyte cell lines and regulation by MAPK and AKT signaling, Murata and colleagues found that apoptosis was induced by decreased MAPK/AKT activity [35]. Moreover, a number of herbal derivatives have also been shown to exhibit anticancer activity via MAPK and/or AKT downregulation. To name a few: jacaranone, a benzoquinone isolated from flowering plant *Pentacalia desiderabilis* was shown to induce apoptosis in melanoma cells via Ros-mediated downregulation of AKT and p38 MAPK activation and exhibit antitumor activity *in vivo* [36]. Baicalin, a flavonoid compound from another flowering plant, was shown to induce apoptosis through downregulation of PI3K/AKT pathway [37]. Icaritin, a derivative from a flowering plant belonging to genus Epimedium, or “horny goat weed” as translated from Chinese, was also found to induce AML cell apoptosis via MAPK/ERK and PI3K/AKT pathways [38]. The role of JNK in apoptosis has also been well documented [34], [39].

It is the most interesting to observe a global collapsing of liver cancer miRNA system in response to *A. cinnamomea* fruiting body extract treatment. To further understand the process, some important issues need to be addressed in the future. For example, what ingredient(s) interact with Drosha and/or Dicer? How the *A. cinnamomea* derivatives enter cancer cells? How these ingredients work synergistically to accomplish a global effect? Future experiments using individual isolates in conjunction with high throughput screening will be helpful to answer at least some of these important questions.

## Supporting Information

Figure S1 **HPLC profile of the** ***A. cinnamomea*** **fruiting body constituents in our AcFBE preparation.** Components of the AcFBE preparation were displayed by HPLC using Antrocamphin A as a reference.
(TIF)

Table S1 **miRNA library statistics of SK-HEP-1.**
(DOCX)

Table S2 **miRNA library statistics of BNL CL.2 normal cell control.**
(DOCX)
