# Supplementary figures and images for: Global Assessment of Antrodia cinnamomea-Induced MicroRNA Alterations in Hepatocarcinoma Cells
Source: PLoS One. 2013 Dec 17;8(12):e82751. doi: 10.1371/journal.pone.0082751 (PMC3866163; doi:10.1371/journal.pone.0082751)

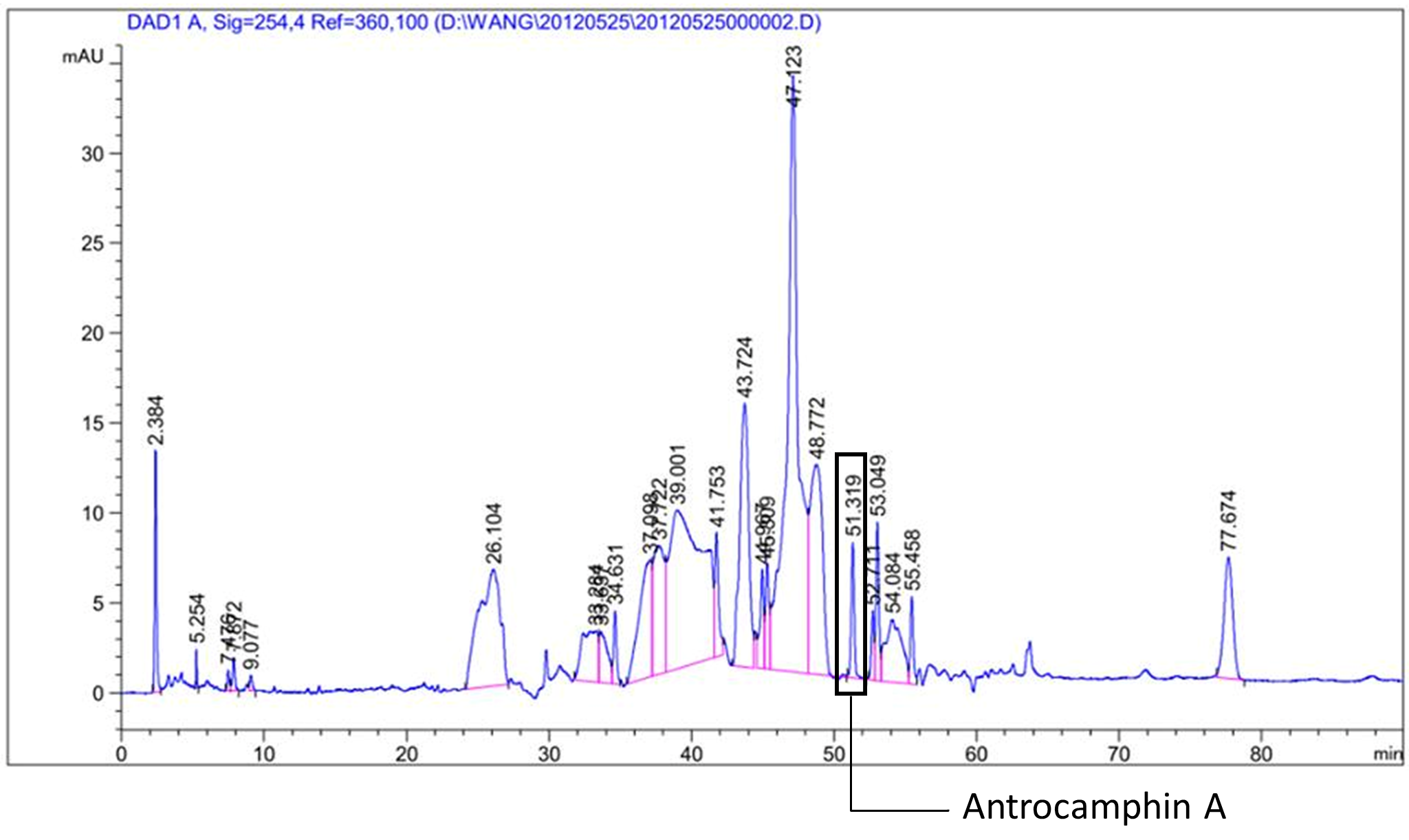

Supplement: Figure S1 — HPLC profile of the A. cinnamomea fruiting body constituents in our AcFBE preparation. Components of the AcFBE preparation were displayed by HPLC using Antrocamphin A as a reference. (TIF) [file pone.0082751.s001.tif]
