# Supplementary material for: Global Assessment of Antrodia cinnamomea-Induced MicroRNA Alterations in Hepatocarcinoma Cells
Source: PLoS One. 2013 Dec 17;8(12):e82751. doi: 10.1371/journal.pone.0082751 (PMC3866163; doi:10.1371/journal.pone.0082751)
Supplement: Table S1 — miRNA library statistics of SK-HEP-1. (DOCX) [file pone.0082751.s002.docx]

**Table S1. miRNA library statistics of SK-HEP-1.**

| SK-HEP-1 libraries | First miRNA dataset  (sequenced by SOLiD 3) | | | | Second dataset  (sequenced by SOLiD 5500xl) | | | |
| --- | --- | --- | --- | --- | --- | --- | --- | --- |
|  | **2U** | **2T** | **4U** | **4T** | **2U** | **2T** | **4U** | **4T** |
| Raw reads | 43,773,943 | 48,929,708 | 48,290,953 | 45,569,187 | 57,341,638 | 43,024,134 | 72,119,641 | 34,570,093 |
| Quality reads | 7,131,622 | 7,692,670 | 11,782,455 | 9,175,263 | 21,069,453 | 17,000,767 | 20,201,916 | 14,109,705 |
| After removal of adapter | 7,078,057 | 7,612,994 | 11,089,337 | 9,114,953 | 20,974,125 | 16,975,664 | 19,908,776 | 14,052,775 |
| Unique reads | 480,109 | 610,931 | 500,792 | 601,772 | 5,254,970 | 4,287,473 | 5,465,387 | 4,087,129 |
| Total reads after removal of polyNs | 7,078,048 | 7,612,928 | 11,089,285 | 9,114,847 | 20,952,538 | 16,961,589 | 19,901,210 | 14,045,912 |
| Total reads after filtering out of reads shorter (16) & longer (30) | 4,369,902 | 3,742,880 | 6,866,734 | 5,361,030 | 6,611,555 | 5,929,923 | 7,805,729 | 5,535,899 |
| Total reads after rRNA filtered | 4,311,686 | 3,664,508 | 6,744,120 | 5,236,424 | 6,527,213 | 5,856,354 | 7,690,495 | 5,445,147 |
| Total reads after removed repeat elements | 4,277,933 | 3,645,236 | 6,675,236 | 5,205,772 | 6,472,614 | 5,825,827 | 7,642,820 | 5,427,037 |
| Total reads after tRNA filtered | 3,361,111 | 3,136,707 | 4,787,456 | 4,801,874 | 3,593,498 | 4,560,955 | 4,920,037 | 4,594,902 |
| Total Reads mapped to miRBase | 1,116,200 | 275,774 | 978,267 | 278,619 | 683,143 | 226,820 | 593,159 | 193,638 |
| Detected number of miRNAs in miRBase | 291 | 228 | 319 | 234 | 317 | 250 | 305 | 250 |
| 2U, 2-hr untreated; 2T, 2-hr treated; 4U, 4-hr untreated; 4T, 4-hr treated. | | | | | | | | |
