# Supplementary material for: Global Assessment of Antrodia cinnamomea-Induced MicroRNA Alterations in Hepatocarcinoma Cells
Source: PLoS One. 2013 Dec 17;8(12):e82751. doi: 10.1371/journal.pone.0082751 (PMC3866163; doi:10.1371/journal.pone.0082751)
Supplement: Table S2 — miRNA library statistics of BNL CL.2 normal cell control. (DOCX) [file pone.0082751.s003.docx]

**Table S2. miRNA library statistics of BNL CL.2 normal cell control.**

| BNL CL.2 libraries | Second dataset  (sequenced by SOLiD 5500xl) | | | |
| --- | --- | --- | --- | --- |
|  | **2U** | **2T** | **4U** | **4T** |
| Raw reads | 12,717,855 | 19,305,589 | 15,242,774 | 14,645,202 |
| Quality reads | 5,933,784 | 10,561,902 | 8,804,857 | 8,881,010 |
| After removal of adapter | 5,364,358 | 9,097,105 | 8,069,891 | 7,195,531 |
| Unique Reads | 1,168,675 | 1,596,097 | 1,684,174 | 1,578,473 |
| Total reads after removal of polyNs | 5,363,831 | 9,096,491 | 8,069,228 | 7,194,853 |
| Total reads after filtering out of reads shorter (16) & longer (30) | 2,632,628 | 4,916,070 | 4,065,713 | 3,576,489 |
| Total reads after rRNA filtered | 2,547,920 | 4,805,092 | 3,938,538 | 3,473,535 |
| Total reads after removed repeat elements | 2,507,871 | 4,639,010 | 3,865,205 | 3,412,932 |
| Total reads after tRNA filtered | 2,351,443 | 4,563,073 | 3,772,782 | 3,357,989 |
| Total reads mapped to miRBase | 1,053,494 | 1,733,757 | 1,484,146 | 1,230,861 |
| Detected number of miRNAs in miRBase | 246 | 254 | 250 | 226 |
| 2U, 2-hr untreated; 2T, 2-hr treated; 4U, 4-hr untreated; 4T, 4-hr treated. | | | | |
